# The Effect of Sharrows, Painted Bicycle Lanes and Physically Protected Paths on the Severity of Bicycle Injuries Caused by Motor Vehicles

**DOI:** 10.3390/safety2040026

**Published:** 2016-12-10

**Authors:** Stephen P. Wall, David C. Lee, Spiros G. Frangos, Monica Sethi, Jessica H. Heyer, Patricia Ayoung-Chee, Charles J. DiMaggio

**Affiliations:** 1 Ronald O. Perelman Department of Emergency Medicine, New York University School of Medicine, New York, NY 10016, USA; 2 Department of Population Health, New York University School of Medicine, New York, NY 10016, USA; 3 Division of Trauma and Surgical Critical Care, Department of Surgery, Bellevue Hospital Center, New York University School of Medicine, New York, NY 10016, USA

**Keywords:** bicyclists, bicycle lanes, geographic analysis, injury severity, trauma

## Abstract

We conducted individual and ecologic analyses of prospectively collected data from 839 injured bicyclists who collided with motorized vehicles and presented to Bellevue Hospital, an urban Level-1 trauma center in New York City, from December 2008 to August 2014. Variables included demographics, scene information, rider behaviors, bicycle route availability, and whether the collision occurred before the road segment was converted to a bicycle route. We used negative binomial modeling to assess the risk of injury occurrence following bicycle path or lane implementation. We dichotomized U.S. National Trauma Data Bank Injury Severity Scores (ISS) into none/mild (0–8) versus moderate, severe, or critical (>8) and used adjusted multivariable logistic regression to model the association of ISS with collision proximity to sharrows (i.e., bicycle lanes designated for sharing with cars), painted bicycle lanes, or physically protected paths. Negative binomial modeling of monthly counts, while adjusting for pedestrian activity, revealed that physically protected paths were associated with 23% fewer injuries. Painted bicycle lanes reduced injury risk by nearly 90% (IDR 0.09, 95% CI 0.02–0.33). Holding all else equal, compared to no bicycle route, a bicycle injury nearby sharrows was nearly twice as likely to be moderate, severe, or critical (adjusted odds ratio 1.94; 95% confidence interval (CI) 0.91–4.15). Painted bicycle lanes and physically protected paths were 1.52 (95% CI 0.85–2.71) and 1.66 (95% CI 0.85–3.22) times as likely to be associated with more than mild injury respectively.

## 1. Introduction

Pedal bicycling improves public health directly by increased physical activity and indirectly by reducing emissions from automotive transportation [1–3]. Despite these benefits, bicyclists incur injuries from falls and collisions with pedestrians, other cyclists, and automobiles. In 2014, nearly 730 pedal bicyclists in the United States (US) were killed in collisions with motor vehicles, a 3% decline from 2013 [4]. Prior to this modest decrease, fatalities had increased by 17% after a trough of 623 fatalities reported in 2010. Seventy-one percent of US bicyclist fatalities occur in urban locations. New York City (NYC) reported the highest number in 2014, with twenty fatalities (2.36 per million population) accounting for 8% of all its traffic related fatalities [4]. Fatality statistics only reflect part of the morbidity and costs to society incurred from bicyclist collisions with automobiles. Nearly 50,000 bicyclists were injured in 2014, a number that has remained relatively stable since 2008. Estimated costs to society from bicycle injuries and deaths exceed 10 billion US dollars per year, in part due to nearly 500,000 emergency department (ED) visits required to care for the injured and the loss of productive life years in a predominantly younger population preferring this transport mode [5].

Prior literature using traffic incident report data ascertained behaviors and environmental factors that influence injury severity among injured bicyclists, including use of helmets and other protective gear, rider adherence to traffic laws and their enforcement, injury mechanisms and locations, and environmental factors including weather, visibility, roadway conditions, and bicycle route design [6–14]; yet a recent Cochrane review found “insufficient evidence to draw any robust conclusions concerning the effect of cycling infrastructure on cycling collisions in terms of severity of injury”, contradicting support for their dissemination [15]. Unfortunately, most estimates found in the literature are limited since many injuries are often understated by traditional surveillance using police accident reports that are often miscoded or lack reliable and valid injury severity scoring [16]. To better understand the burden of non-fatal bicyclist injuries, researchers have merged in-hospital data with pre-hospital injury reporting [17–24]. These data corroborate patterns of behavior and injury mechanisms that impact injury severity from bicycle–automobile collisions while suggesting that bicycle route infrastructure protects riders.

In 2014, NYC launched ‘Vision Zero’, a multidisciplinary traffic safety action plan with a strong government commitment aimed at eliminating traffic-related deaths and serious injuries [25]. Severe traffic-related injuries and deaths were no longer deemed ‘acceptable’ circumstances of urban living. As part of its Vision Zero Initiative, NYC focused significantly on bicycle safety [25] through construction of a comprehensive network of bicycle routes that includes “sharrows” (i.e., road lanes shared with bicycles and cars), bicycle lanes demarcated with painted lines (i.e., painted bicycle lanes) and over 30 miles of protected paths alongside streets having physical barriers that separate automobile traffic from bicyclists (i.e., physically protected paths) [26,27]. We sought to assess whether sharrows, painted bicycle lanes, and physically protected paths reduce injury occurrence and severity among urban bicyclists. Our hypotheses were that painted bicycle lanes and physically protected paths are associated with decreased injury occurrence, and that among a population of urban bicyclists who collided with automobiles, injury severity was reduced when sharrows, painted bicycle lanes, or physically protected paths were available compared to when no such protections were present at collision locations [28,29].

## 2. Materials and Methods

### 2.1. Study Design and Setting

We analyzed data collected for a prospective study that recruited injured pedal bicyclists presenting to Bellevue Hospital, an urban Level-1 trauma center in NYC, United States of America, with a catchment area encompassing Southern Manhattan and Western Brooklyn. The study initially prospectively recruited a cohort of 1471 pedestrians and bicyclists who were struck by motor vehicles between December 2008 and June 2011 [18]. The study was extended to recruit an additional 706 bicyclists who were injured from falls and collisions with pedestrians, bicycles, or motor vehicles between February 2012 and August 2014 [22]. We then screened both datasets to identify bicyclists injured from motor vehicle collisions to have comparable data for analysis. Institutional review boards from NYU School of Medicine and Bellevue Hospital approved this work.

Research protocols are described in detail elsewhere [19]. Crash scene data were obtained through patient interviews and when available, interviews with emergency medical services (EMS) providers, police, and witnesses in the ED to corroborate findings or obtain data when patients were obtunded. We abstracted injury data from medical records. Alcohol use was defined as blood alcohol concentration greater than 0.01 g/dL or recent consumption prior to the incident. Injury Severity Score (ISS) was categorized according to US National Trauma Data Bank (NTDB) definitions—none or mild (0–8), moderate (9–15), severe (16–25), critical (>25)—coded by a trauma surgeon, which was then dichotomized into none or mild (0–8) versus moderate, severe, or critical injury (>8). Scene variables included patient demographics, riding behaviors, helmet use, traffic law compliance, weather conditions, street classification, and crash mechanism (Table 1).

### 2.2. Geographic Identification of Bicycle Lanes and Use

To reduce bias from self-reporting (e.g., concussed patients), we classified whether bicyclists were injured on streets with sharrows, painted bicycle lanes or physically protected paths using address data from EMS records. We geocoded incident locations with bicycle route GIS data (shapefile = ESRI) provided by the NYC Department of Transportation (DOT) [29]. When incidents occurred at intersections, we used the first street as the travel route and the second street as the crossing. We verified bicycle sharrow/lane/path existence using incident dates from the datasets and dates of bicycle sharrow/lane/path installation from the NYC DOT Shapefile. This algorithm accounted for temporal changes in bicycle lane and path availability during the study period.

### 2.3. Modeling Injury Risk

We fit two separate over-dispersed quasi-Poisson models to assess the effect of physically protected paths and painted bicycle lanes on bicycle crash injury count per month during the study period:

crash count in month=β0+β1×path/lane+β2×time+β3×path/lane×time+log(pedestrian count)

where, “path/lane” refers to the crash occurring on a Geographic Information System (GIS) determined segment of road designated to be a physically protected path or painted bicycle lane (depending on the model), “time” refers to time period, coded as 0 for the pre-lane or path period which varied by road segment, and “pedestrian count” [30] is included as an estimated population offset or proxy for bicycle counts, allowing the exponentiated coefficients to be interpreted as incidence density ratios. Pedestrian counts were determined to be a reasonable proxy given lack of bicycle count data (see limitations section for a complete discussion). Sharrows and painted bicycle lanes were merged into a composite category due to low numbers of incidents occurring nearby Sharrows.

In the model framework:

- The intercept (*β*0) is the risk when both path/lane and intervention period are set to zero, i.e., the baseline rate in the non-bicycle route areas before the intervention
- The *β*1 coefficient is the effect when path or lane is set to 1 and the time period is set to zero
- *β*2 is the post-intervention effect when path or lane is held to zero, from which we can calculate the rate in the non-path/lane areas following the intervention time period, which is *β*0 + *β*2
- The interaction term *β*3 is the effect when both bicycle path or lane and intervention time period are set to 1. The coefficient for the interaction term for time period and intervention status is interpreted as a measure of the change in incidence density ratios from the pre-intervention period to the post-intervention period

A full set of R codes for the data preparation and analysis for the model is available as supplemental material.

### 2.4. Individual Data Analysis of Injury Severity

We used adjusted multivariable logistic regression to model the effect of bicycle route availability (sharrows, painted bicycle lanes, physically protected paths, and none) from other predictors on ISS categories. Variables were included based on known epidemiology of traffic injuries or hypothesized associations with outcomes (Table 1). Odds ratios with 95% CIs were reported after adjustment with multiply imputed data to account for missing data. We used Markov Chain Monte Carlo methods for multiple imputation to generate 20 multiply-imputed datasets, each analyzed independently and combined using Rubin’s rules to appropriately account for within- and between-dataset variance [31]. The multiple imputation model included all relevant variables in the dataset for which there were no missing data including secondary outcomes (e.g., hospital admission) and demographics. Logistic regression model fit was assessed using the methods proposed by Hosmer and Lemeshow [32]. Statistical analyses were performed using Stata Version 13 (College Station, TX, USA).

### 2.5. Geographic Clustering of Injury Severity

We used geographic analysis to identify significant clusters of high and low ISS [33,34]. First, we determined the distance band within which clustering of values was highest (i.e., the first and maximal peaks of statistically significant spatial autocorrelation). Then, we performed clustering analysis within this distance band using Anselin Local Moran’s I statistic with inverse Manhattan distance to moderate the influence of nearby events, and row standardization to account for non-uniformity of event density. Finally, we mapped the location of high and low ISS clusters onto the Bicycle Route Shapefile (We provide a thorough methods description in Appendix A).

## 3. Results

The first study enrolled 1471 vulnerable roadway users of whom 382 were bicyclists injured by motor vehicles. The second study enrolled 706 injured bicyclists of whom 460 collided with motor vehicles, totaling 842 cases. Both studies captured over 95% of eligible patients presenting to Bellevue Hospital. Of these, one lacked ISS, one lacked EMS information, and one lacked alcohol use, leaving 839 for analysis. Missing elements for imputation ranged between 1% and 55% of cases (Table S1). Of the 839 patients, 698 (83%) had no or mild (ISS 0–8), 90 (10%) had moderate (ISS 9–15), 24 (2.9%) had severe (ISS 16–25), and 27 (3.2%) had critical (>25) injuries; five patients died.

### 3.1. Injury Risk Analysis

**Figure 1:**
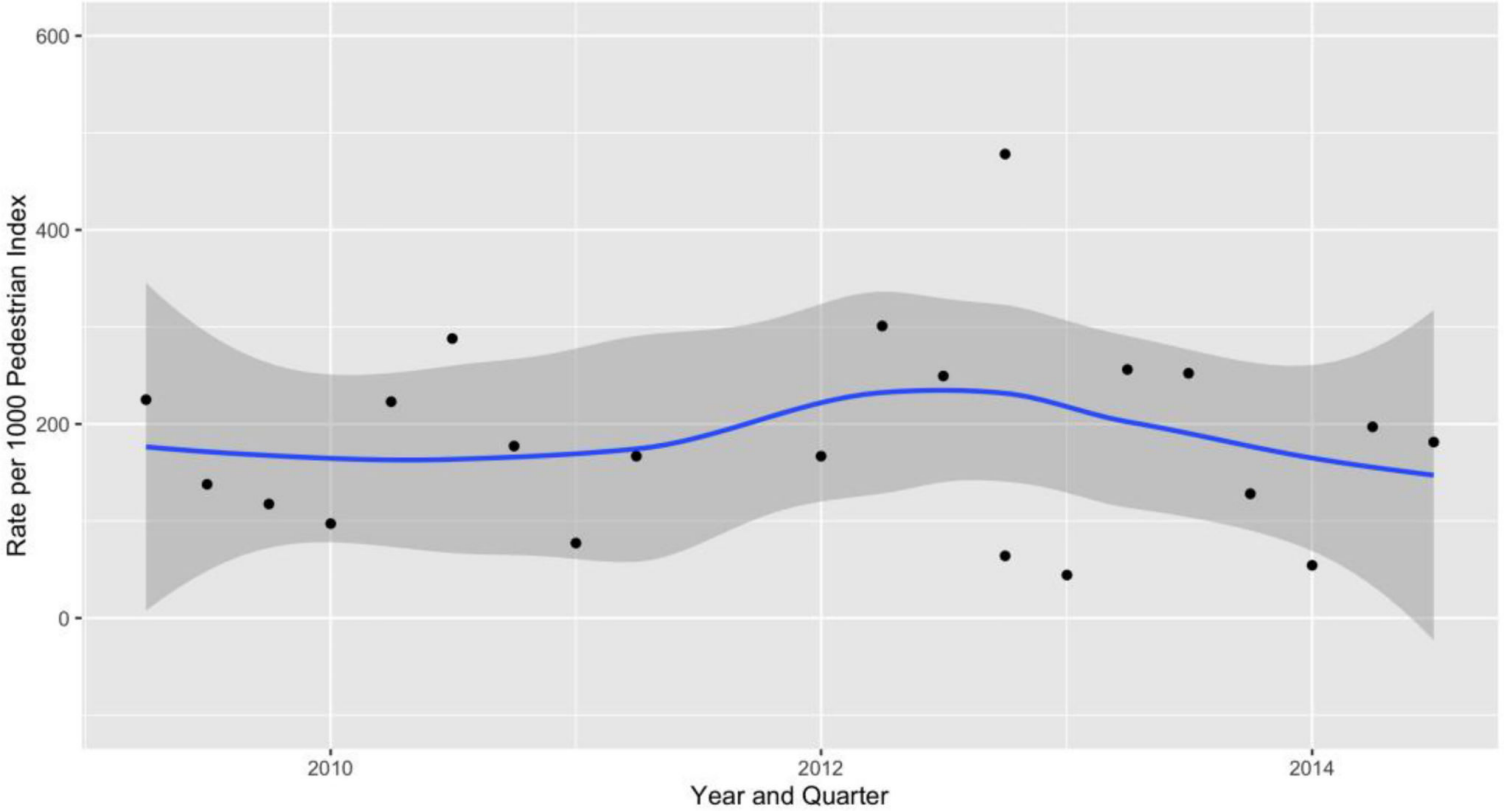
Bicycle Injuries per 1000 Pedestrians Count by Year and Quarter. New York City, Bellevue Hospital Catchment Area, 2008–2014.

Visual inspection of a loess smoothed plot of quarterly injury counts indicated a decreasing trend in the latter third of the study period (Figure 1).

The results of the risk modeling indicate that physically protected paths and painted bicycle lanes were associated with a dramatic decrease in the number of bicycle injuries presenting to our Level-1 trauma center. Physically protected paths were associated with approximately 23% fewer injuries compared to non-bicycle route locations, though this result was not statistically significant (Incidence Density Ratio (IDR) = 0.76, 95% CI 0.00–20.61). Painted bicycle lanes were associated with almost 90% fewer injuries (IDR = 0.09 95% CI 0.02–0.33). The interaction term for the post physically protected path time period indicated a safety benefit of almost 73% fewer injuries (IDR = 0.22, 95% CI 0.01–492), although the data were unable to demonstrate statistical significance. The interaction term for painted bicycle lanes indicated a non-statistically significant increase in injuries of 30% (IDR = 1.3 95% CI 0.9–1.8).

Unadjusted analysis of the primary variable of interest (bicycle route type) and potential confounders are presented in Table 1 using ISS (0–8, >8) as the outcome. Incidents occurring at sharrows, painted bicycle lanes, and physically protected paths had approximately 4% more severely injured riders than expected. Those having no designated bicycle route available had 12% less severely injured riders; however, these results were not statistically significant. Table 2 demonstrates that ISS is predictive of injury outcomes, with ISS >8 being associated with transport by EMS, having a Glasgow Coma Scale (GCS) score <15, and being admitted or dying from the incident. The Adjusted multivariable logistic regression model (Table 3) revealed that holding all else equal, sharrows were associated with having 94% increase in log odds of incurring more than mild injury compared to having no bicycle route available (Adjusted Odds Ratio (AOR) 1.94 95% Confidence Interval (CI) 0.91–4.15). Proximity to painted bicycle lanes and physically protected paths was associated with having 52% (AOR 1.52 95% CI 0.85–2.71) and 66% (AOR 1.66 95% CI 0.85–3.22) increases in the log odds of having more than a mild injury respectively. Results from the adjusted multivariable logistic regression were consistent with the stratified analysis and unadjusted odds ratios. Only sharrows in the unadjusted model, and age and delivery worker from the adjusted model were statistically significant.

### 3.2. Geographic Analysis

**Figure 2:**
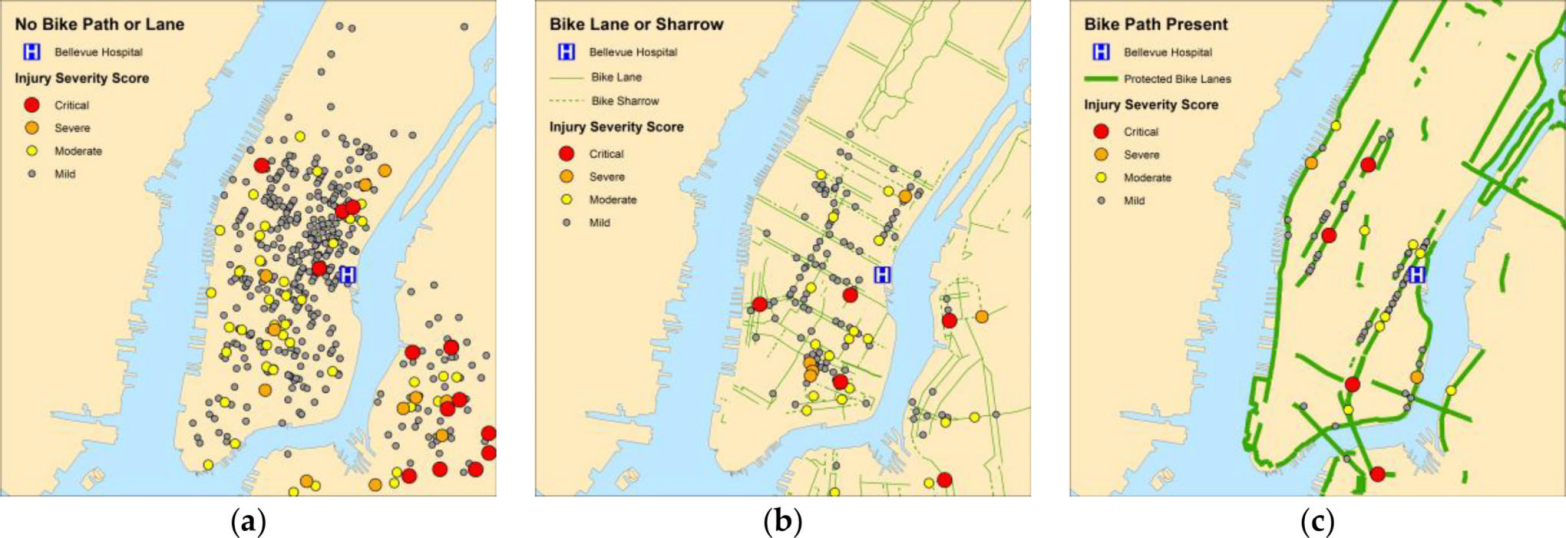
Comparison of ISS based on presence or absence of bicycle routes. Legend: Maps of injury severity scores (ISS) for bicyclists struck by motor vehicles based on (**a**) absence of bicycle lane/path; (**b**) presence of sharrow or painted bicycle lane; and (**c**) presence of physically protected path. ISS is color-coded based on severity: critical (red), severe (orange), moderate (yellow), and mild (gray).

We examined the proportion of greater than mild injuries (ISS >8) by NYC neighborhood (Figure 2). Aside from numerous severe and critical injuries that occurred at the edge of Bellevue Hospital’s EMS catchment area outside of Manhattan, the proportion of injuries classified as ISS >8 occurring without a painted bicycle lane, sharrow or physically protected path were relatively low in mid-town and downtown Manhattan. However, for injuries in sharrows or painted bicycle lanes, the proportions of ISS >8 injuries were higher for the eastern-most neighborhood of downtown Manhattan. Except for the western downtown neighborhoods that had few injury counts, we also found that the proportions of ISS >8 injuries in physically protected paths were higher for downtown and midtown Manhattan. Of note, some physically protected paths had no injuries or none greater than mild during the study period.

### 3.3. Geographic Clustering of Injury Severity Scores

**Figure 3:**
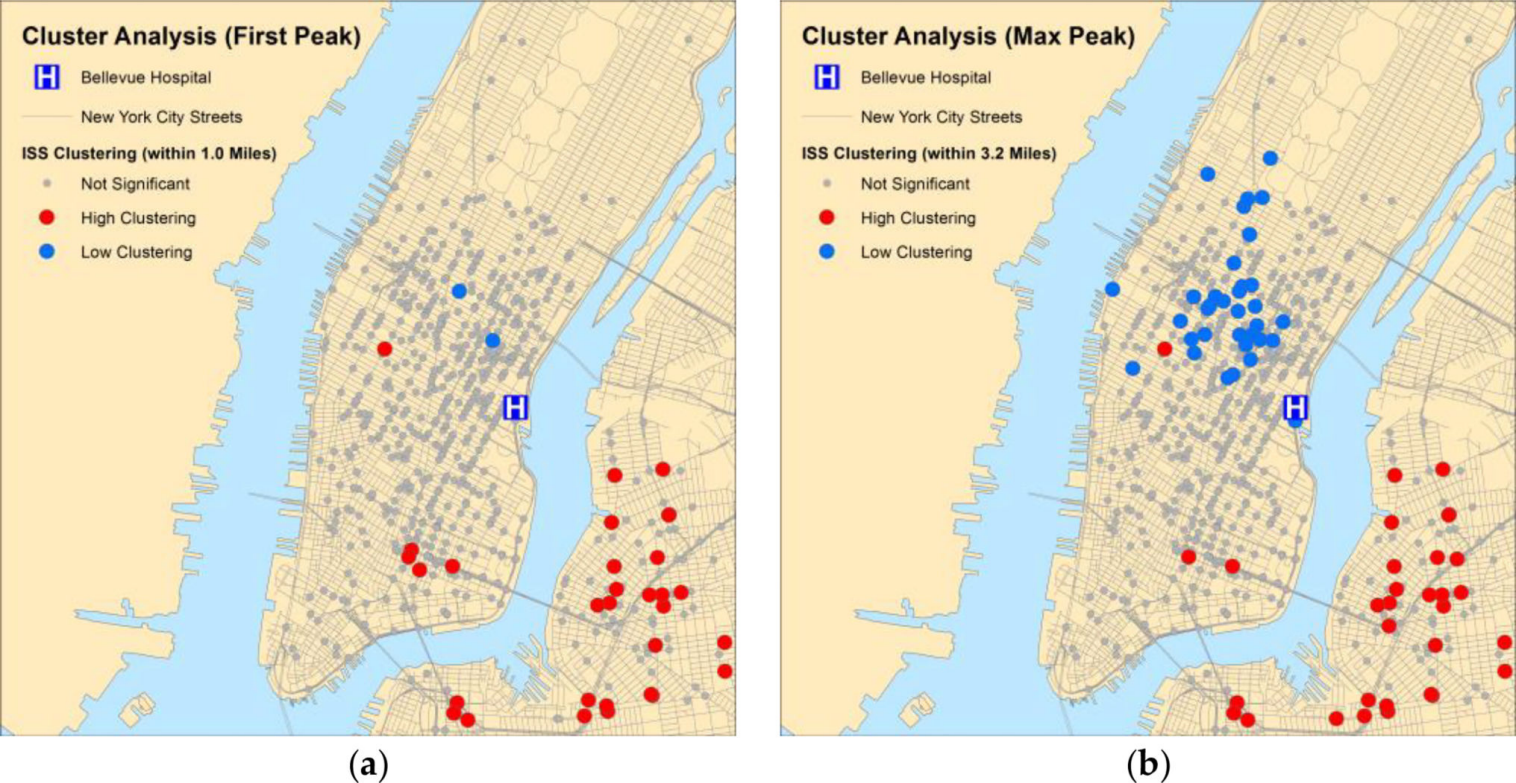
Clustering of high ISS and low ISS among bicyclist trauma incidents. Legend: Results of the clustering analysis of high injury severity scores (ISS) and low ISS based on Anselin Local Moran’s I analysis. The above depicts statistically significant high-to-high clusters and low-to-low clusters for bicyclist injuries using the distance band at the (**a**) first and (**b**) maximal peaks of significance for incremental spatial autocorrelation.

Incremental spatial autocorrelation analysis identified the first and maximally statistically significant peaks at 1.0 and 3.2 miles (Figure S1). We used these distances to define the limits within which injury events influenced each other. Cluster analysis demonstrated locations having high and low ISS. Most low ISS clusters were found in midtown Manhattan. Most high ISS clusters were located in the periphery of Bellevue’s catchment area or near bridge and tunnel crossings (Figure 3).

## 4. Discussion

We find in our analyses that the role of sharrows, painted bicycle lanes, and physically protected paths is complex; all of these designated bicycle routes were associated with a decreased risk of injury; however, when injuries do occur, they tended to be more severe. In our logistic regression model, we saw a 94% increase in log odds of having increased injury severity when bicyclists collided with a motor vehicle at locations having sharrows, a 52% increase in painted bicycle lanes, and a 66% increase in physically separated paths. Our geographic analysis identified many high ISS clusters near bridges and tunnel portals, which tend to have sharrows, painted bicycle lanes, and physically protected paths present. The low ISS clusters were located mostly in midtown Manhattan, areas having higher traffic density. Some physically protected paths have no injuries or none greater than mild severity, suggesting that there may be unique designs or circumstances that may be extrapolated to other bicycle routes where incidents are occurring at rates greater than by chance alone.

**Figure 4:**
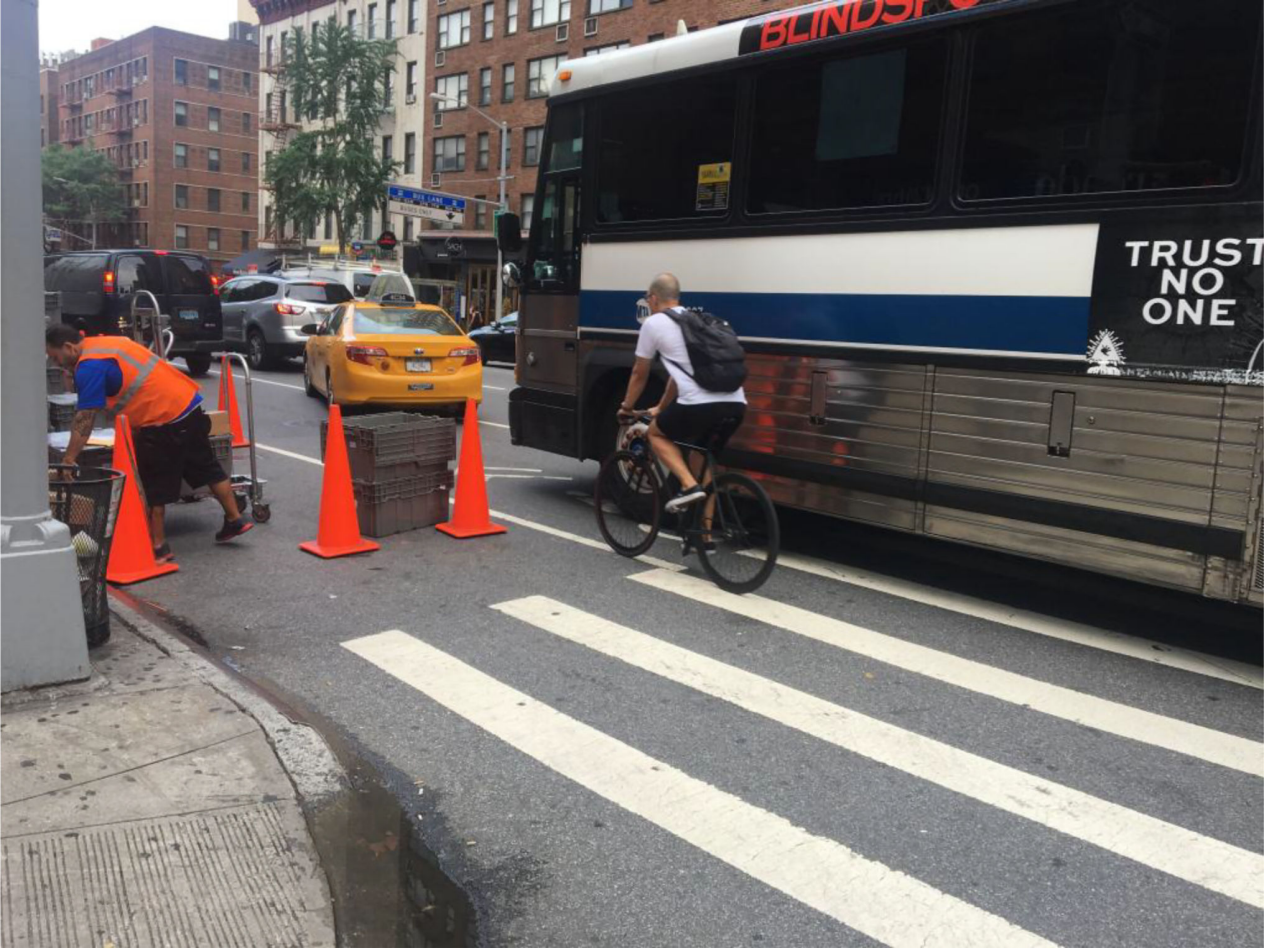
Bicyclist navigating a sharrow. This street location in New York City corresponds to the critical hotspot on Second Avenue (Figure 2b). Sharrow riders merge with automobiles making left hand turns. Bicyclists are instructed to ride in a shared lane with traffic, but often they pass automobiles rather than using the lanes as intended. Automobile drivers are unaccustomed to having bicyclists in their left blind spot. Qualitative investigations of scene locations may improve understanding of why design features are associated with increased ISS and frequency of collisions.

Our results suggest that increased injury severity is most severe with shared bicycle routes. In NYC, sharrows are shared bicycle routes with cars, ideally meant for low speed traffic, whereby a bicycle is meant to have its own space, as if it were a car in that lane; unfortunately, bicyclists and automobile drivers often navigate side by side or weave through traffic at higher speeds than intended (Figure 4).

The current state of evidence reflects some of the complexity of our findings. Increased injury severity has been shown to occur when bicyclists are injured on shared routes with walkers and joggers [17]. While the recent Cochrane review found inconclusive evidence to justify separation of bicycles from automobile and pedestrian traffic [15], others have found reduced injury incidence and severity from such separation [12,28]. The 2014 NYC DOT report that analyzed the New York Police Department Accident Investigation Squad Traffic Accident Management System data found that physically protected paths reduced bicycle crash injuries by 17% despite dramatically increasing volumes [28]. Though physically protected paths may have decreased the absolute number of injuries when injuries did occur, our data suggest that they seemingly are of higher severity. Painted bicycle lanes similarly have been found to reduce injury risk, however, in a study of the effectiveness of various safety countermeasures and street designs installed in NYC from 1990 to 2008, painted bicycle lanes were associated with approximately 13% increased risk in mid-block injuries [35]. A separate study with similar data looked explicitly at painted bicycle lanes, with the additional finding that perhaps the most important driver of injury occurrence is daytime population density [36].

Our findings about injury risk are supported by other investigations. From prior study estimates, there are well over twice as many bicyclists on painted bicycle lanes than on unmarked streets, and when rates of bicycle use are closely measured and comparisons methodologically rigorous, bicycle lanes are safer than non-lanes with approximately 28% fewer crashes and injuries [37]. Another analysis confirmed when adjusting for potential confounders and underlying exposure in terms of average annual daily traffic, bicycle lanes are associated with a decreased number of injuries [38]. Our injury risk analysis corroborates these findings. However, these studies evaluated frequency of injuries, not severity. Injury severity is important to consider when the goal is reducing bicyclist mortality and morbidity, and this data is readily available in EMS and hospital data [16]. With this additional information, data suggest that while overall rates of injury may be declining, severity of injuries incurred may be higher, assuming our trauma center’s experience is generalizable to the overall population of injured bicyclists presenting to NYC area trauma centers. Our findings warrant further study based on a more comprehensive analysis of bicyclist trauma throughout NYC and in other urban areas where bicycle route infrastructure warrants examination of separating bicyclists from motor vehicles.

### Limitations

Our study is limited by data that only included injured riders who were seen at Bellevue, whether transported by EMS or arrived by other means. It is conceivable that some riders injured within Bellevue’s catchment area may have presented to other area hospitals. Since Bellevue is the designated Level-1 trauma center in the area, it is likely that the sample has greater injury severity than the total sample of riders. In support of this possibility, we found some higher ISS clusters occurred in the periphery of Bellevue’s catchment area. Furthermore, many injured riders do not present to EDs or even seek care, and the sample does not capture any near-misses, which would identify infrastructure issues worth addressing prior to resulting in injury. Nonetheless, given Vision Zero’s emphasis that no fatality is acceptable, the data illuminate problem areas which may be targeted for infrastructure modifications to prevent future events [25,39]. Precisely determining how changes in infrastructure affect injury severity requires improved surveillance.

To determine whether bicyclist injuries occurred on sharrows, painted bicycle lanes, or physically protected paths, we primarily relied on matching EMS address data with geographic data. However, it may be that the bicyclist was on a street with an available painted bicycle lane or physically protected path, but the individual was not actually riding in it. We favored using this method over self-report as we found that many individuals who reported being on a physically protected path or painted bicycle lane were not geographically near any known bicycle path or lane. This discrepancy may be from transient concussive states or simply misunderstanding what constitutes a painted bicycle lane or physically protected path. Aggregating sharrows and dedicated bicycle lanes may have introduced bias in the injury risk and GIS models; however, their aggregation was required due to low numbers of incidents in these locations. Our analysis of high and low ISS clusters does not account for other variables that might influence injury severity.

Perhaps the most challenging aspect of these analyses involved determining an appropriate data source to control for the population at risk. That there are bicycling injuries in bicycle paths and lanes is a tautology. That is where the bicyclists are. The challenge is to measure changes in ridership when those paths and lanes are installed. In this case, there were few or no sources for such data. As a proxy measure, we chose pedestrian count data collected by the NYC DOT. These are clearly not ideal, but they do provide some level of context for the injury risk analyses. We evaluated the correspondence of pedestrian count data locations to bicycle crash locations by matching both data sets on cross streets. We found our bicycle crash cross street locations were matched to pedestrian count locations in Manhattan in 61.9% of the data. There were partial matches, indicating somewhere on the same street but a different avenue for 17.6% of the data. We believe that this indicates that the pedestrian count data is a reasonable, though clearly not a perfect, approximation for bicycling activity in the area of the bicycle crashes.

## 5. Conclusions

In dense urban environments, sharrows, painted bicycle lanes, and physically protected paths were associated with fewer injuries, but when injuries occur, they tended to be more severe. In our study, some physically protected paths lacked any injuries at all, suggesting heterogeneity of risk, such that their designs and use may be informative to improve bicycle route infrastructure in other locations where injury risk and severity are greater than expected. Precisely determining how changes in infrastructure influence injury severity requires improved surveillance and better data sources with improved estimates of the population at risk. Future studies with more data from multiple trauma centers that incorporate additional information on the cycling experience of injured patients are needed to further advance and corroborate these findings.

## Supplementary Material

Supplemental Material Table S1: Missing Data for Variables included in the Logistic Regression, Figure S1: Incremental spatial autocorrelation to determine clustering distance band.

## Figures and Tables

**Table 1:** Stratified analysis of behavior and environment variables by injury severity score (ISS).

	ISS *≤*8 (None or Mild)		ISS >8 (Moderate, Severe or Critical)	

Variable	*n* (%)	95% CI	*n* (%)	95% CI
Bicycle Route				

None	486 (72%)	69%–76%	78 (60%)	51%–69%
Sharrow	38 (5.6%)	4.0%–7.7%	13 (10%)	5.4%–16%
Painted Bicycle Lane	91 (14%)	11%–16%	22 (17%)	11%–24%
Physically Protected Path	58 (8.4%)	6.6%–11%	17 (13%)	7.8%–20%

Gender				

Male	589 (84%)	81%–87%	119 (84%)	77%–90%
Female	109 (16%)	13%–19%	22 (16%)	10%–23%

Age				

<18	31 (4.5%)	3.0%–6.3%	4 (3.0%)	0.78%–7.1%
18–55	622 (89%)	87%–91%	114 (81%)	73%–87%
>55	45 (6.5%)	4.7%–8.5%	23 (16%)	11%–23%

Ethnicity				

Non-Latino White	256 (37%)	33%–41%	73 (52%)	43%–60%
Black	92 (13%)	11%–16%	8 (5.7%)	2.5%–11%
Latino	247 (36%)	32%–39%	39 (28%)	20%–36%
East Asian	67 (9.6%)	7.6%–12%	16 (11%)	6.6%–18%
South Asian	15 (2.2%)	1.2%–3.5%	3 (2.1%)	0.44%–6.1%
Other	18 (2.2%)	1.5%–4.1%	2 (1.2%)	0.17%–5.0%

Alcohol Use				

No	663 (95%)	93%–96%	119 (84%)	77%–90%
Yes	35 (5.0%)	3.5%–6.9%	22 (16%)	10%–23%

Bicycle Share				

No	346 (95%)	92%–97%	83 (97%)	90%–99%
Yes	19 (5.0%)	3.2%–8.0%	3 (3.0%)	0.73%–9.9%

Wore Helmet				

No	454 (66%)	62%–70%	96 (70%)	62%–76%
Yes	234 (34%)	30%–38%	41 (30%)	22%–38%

Delivery Worker				

No	421 (62%)	58%–65%	114 (84%)	77%–90%
Yes	263 (38%)	35%–42%	21 (16%)	10%–23%

Self Reported Speed				

<5 mph	66 (21%)	16%–25%	18 (28%)	17%–40%
5–15 mph	230 (72%)	67%–77%	39 (60%)	47%–72%
>15 mph	24 (7.0%)	5.0%–11%	8 (12%)	5.5%–23%

Hit by Turning Vehicle				

No	230 (40%)	36%–45%	51 (55%)	44%–65%
Yes	339 (60%)	55%–64%	42 (45%)	35%–56%

Distracted Riding (cell phones, audio equipment, etc.)				

No	616 (90%)	88%–92%	116 (91%)	84%–95%
Yes	68 (10%)	8.0%–12%	12 (9%)	5.0%–16%

Salmoning (riding against traffic)				

No	590 (92%)	90%–94%	102 (89%)	81%–94%
Yes	51 (8.0%)	6.0%–10%	13 (11%)	6.0%–18%

Motor Vehicle Type				

Passenger Car	258 (42%)	38%–46%	54 (49%)	39%–58%
Taxi	261 (42%)	38%–46%	26 (23%)	16%–32%
SUV, Van, or Truck	98 (16%)	13%–19%	31 (28%)	20%–37%

Road Condition				

Normal	610 (89%)	86%–91%	122 (90%)	84%–95%
Wet or Iced	75 (11%)	8.7%–14%	13 (10%)	5.2%–16%

At Stop Sign				

No	652 (98)	96%–99%	117 (94%)	89%–98%
Yes	15 (2.0%)	1.3%–3.7%	7 (6.0%)	2.3%–11%

At Traffic Signal				

No	320 (50%)	46%–54%	44 (38%)	29%–47%
Yes	322 (50%)	46%–54%	73 (62%)	53%–71%

Daylight Condition				

Daylight	221 (68%)	62%–73%	26 (50%)	36%–64%
Night	106 (32%)	28%–38%	26 (50%)	36%–64%

A.M. Rush Hour				

No	639 (93%)	91%–95%	127 (91%)	85%–95%
Yes	50 (7.0%)	5.0%–10%	12 (8.0%)	5.0%–15%

P.M. Rush Hour				

No	592 (86%)	83%–89%	122 (88%)	81%–93%
Yes	96 (14%)	11%–17%	17 (12%)	7.3%–19%

Road Classification				

Local Street	326 (75%)	70%–79%	58 (67%)	56%–76%
Avenue or Two Way Arterial	110 (25%)	21%–30%	29 (33%)	24%–44%

**Table 2:** Stratified analysis of process and outcome variables by ISS categories.

	ISS *≤*8 (None or Mild)		ISS >8 (Moderate, Severe or Critical)	

Variable	*n* (%)	95% CI	*n* (%)	95% CI
Brought in by EMS				

No	65 (9.0%)	7.0%–12%	2 (1.0%)	0.17%–5.0%
Yes	633 (91%)	88%–93%	139 (99%)	95%–100%

GCS <15				

No	654 (95%)	93%–96%	104 (76%)	68%–83%
Yes	37 (5.0%)	3.8%–7.3%	33 (24%)	17%–32%

Admitted or Died				

No	610 (87%)	85%–90%	23 (16%)	11%–23%
Yes	88 (13%)	10%–15%	118 (84%)	77%–89%

**Table 3:** Logistic Regression Modeling Injury Severity Score Categories 1.

Unadjusted Model	Odds Ratio	*p* Value	95% CI
Sharrow	2.02	0.040	1.03–3.94
Painted Bicycle Lane	1.50	0.130	0.89–2.53
Physically Protected Path	1.79	0.052	0.99–3.21
Adjusted Model Sharrow	1.94	0.086	0.91–4.15
Painted Bicycle Lane	1.52	0.159	0.85–2.71
Physically Protected Path	1.66	0.136	0.85–3.22
Female	0.68	0.172	0.39–1.18
Age 18–55	0.48	0.010	0.26–0.84
Alcohol Use	1.94	0.235	0.65–5.81
Bicycle Share	0.90	0.893	0.21–3.92
Wore Helmet 2	0.93	0.731	0.60–1.44
Delivery Worker	0.35	0.000	0.21–0.61
Bicycle Speed 5–15 mph	0.77	0.415	0.41–1.45
Bicycle Speed >15 mph	1.37	0.633	0.37–5.12
Hit by Turning Vehicle	0.78	0.471	0.39–1.54
Distracted Riding	0.82	0.603	0.38–1.74
Salmoning	1.25	0.528	0.62–2.54
Hit by Taxi	0.59	0.068	0.34–1.04
Hit by SUV, Van, or Truck	1.59	0.102	0.91–2.78
Wet or Iced Road	1.09	0.819	0.53–2.25
Hit at Intersection 3	1.47	0.102	0.93–2.34
Hit at Night	1.44	0.481	0.52–4.00
Hit During A.M. Rush	1.14	0.747	0.51–2.57
Hit During P.M. Rush	0.97	0.932	0.49–1.91
Hit on Avenue or Two Way Artery	1.27	0.462	0.67–2.42

1 ISS was defined as four categories of ISS—Mild 0–8, Moderate 9–15, Severe 16–25, Critical >25—and then dichotomized into 0–8 (reference) or >8;

2 Helmet protection effect was likely attenuated from including all injuries; analysis on only those having head injury yields protective benefit [22];

3 At intersection includes those incidents that occurred at a traffic signal or stop sign. Models run on multiply imputed data to preserve all 839 records and outcomes within each ISS category. The Hosmer–Lemeshow goodness of fit statistic, run on the first of 20 imputed data sets, was 0.415, indicating good model fit.

## Abbreviations

US: United States
NYC: New York City
Bellevue: Bellevue Hospital
AOR: Adjusted Odds Ratio
CI: Confidence Interval
ED: Emergency Department
EMS: Emergency Medical Services
GCS: Glasgow Coma Scale
ISS: Injury Severity Score
NTDB: National Trauma Data Bank
GIS: Geographic Information System
DOT: Department of Transportation
IDR: Incidence Density Ratio

## Appendix A

### Geospatial Analysis

The concept of identifying statistically significant clusters using geospatial analysis has been previously well described [40]. If injury severity was determined by a random process, then there would not be a systematic clustering or dispersion of the injury severity scores (ISS), which was the primary outcome in our study. To identify statistically significant clustering of ISS, we used spatial analysis tools in ArcGIS. This clustering analysis would identify areas where several high ISS or low ISS were bunched together in a pattern that would not be expected by a random process. The first step in this analysis is to define its parameters, the most important being the distance band within which the analysis would take place. Essentially, this means the distance within which injury events would influence each other.

For instance if the distance band was a mile, then each incident would be evaluated in the context of the injury severity of other events within a mile. If a disproportion number of the other events had high ISS, then it would suggest possible clustering of high ISS. This distance band is identified using Global Moran’s I. It is a statistical test which identifies the amount of spatial autocorrelation within a specified distances [41]. Spatial autocorrelation is the measure of how data values, such as ISS are clustered together in space (positive spatial autocorrelation) or dispersed (negative spatial autocorrelation). The analysis scans through a range of possible distances to identify the distance band at which clustering is the highest. We show these results graphically using z-scores for the significance of the spatial correlation based on a range of distance bands (Figure S1).

We evaluated both the first statistically significant peak of spatial autocorrelation and the maximal peak in order to see whether using a tight versus wide distance band had any effect on the geographic analysis. We then used Anselin Local Moran’s I statistic using these two specified distances bands as parameters [42]. This analysis identifies the statistically significant hot spots and cold spots with a given set of weighted features. The spatial tool calculates a value for the test statistic along with a z-score and *p*-value. A positive value for the statistic means that neighboring events to a given incident have similarly high or low ISS and thus values are clustering, i.e., a high ISS event that is surrounded by other high ISS events. A negative value for the statistic would mean that the event would be an outlier, i.e., a high ISS is surrounded by low ISS events. In our analysis, we only reported statistically significant clusters of high and low ISS and disregarded the identified outliers.

In all of our clustering analyses, we used Manhattan distances, which means that a grid-like pattern was assumed. Distances between two points were thus calculated by taking the sum of horizontal (x) and vertical (y) differences. As our study was performed in New York City, this was an appropriate assumption. In addition, because event density is influenced by the density of bicyclists at risk for injury and the fact that we analyzed injuries that were treated at a single hospital, the geographic distribution of events varied. To account for this variation, we used row standardization to mitigate any bias from our sampling design. Finally, we used inverse distance to specify how the ISS of different events were related to each other. Thus, other events that was located further away but still within the distance band was assigned less weight than events that occurred closer to the geographic location being analyzed [43].
