# Supplementary material for: The Effect of Sharrows, Painted Bicycle Lanes and Physically Protected Paths on the Severity of Bicycle Injuries Caused by Motor Vehicles
Source: Safety (Basel). Author manuscript; Available in PMC 2018 Mar 19. (PMC5858726; doi:10.3390/safety2040026)
Supplement: Supplemental Material — Table S1: Missing Data for Variables included in the Logistic Regression, Figure S1: Incremental spatial autocorrelation to determine clustering distance band. [file NIHMS926676-supplement-Supplemental_Material.docx]

Supplementary Materials: The Effect of Sharrows, Painted Bicycle Lanes and Physically Protected Paths on the Severity of Bicycle Injuries Caused by
Motor Vehicles

Stephen P. Wall, David C. Lee, Spiros G. Frangos, Monica Sethi, Jessica H. Heyer,
Patricia Ayoung-Chee and Charles J. DiMaggio

**Table S1.** Missing Data for Variables included in the Logistic Regression.

| **Variable** | **Number of Observations** | **Percent Missing of *n* = 839** |
| --- | --- | --- |
| Bicycle Route | 803 | 4% |
| Gender | 839 | 0% |
| Age | 839 | 0% |
| Alcohol Use | 839 | 0% |
| Bike Share | 451 | 46% |
| Wore Helmet | 825 | 2% |
| Delivery Worker | 819 | 2% |
| Self Reported Speed | 385 | 54% |
| Hit by Turning Vehicle | 662 | 21% |
| Distracted Riding | 812 | 3% |
| Salmoning | 756 | 10% |
| Motor Vehicle Type | 728 | 13% |
| Road Condition | 820 | 2% |
| At Stop Sign | 791 | 6% |
| At Stop Light | 759 | 10% |
| Daylight Condition | 379 | 55% |
| AM Rush Hour | 828 | 1% |
| PM Rush Hour | 827 | 1% |
| Road Type | 523 | 38% |


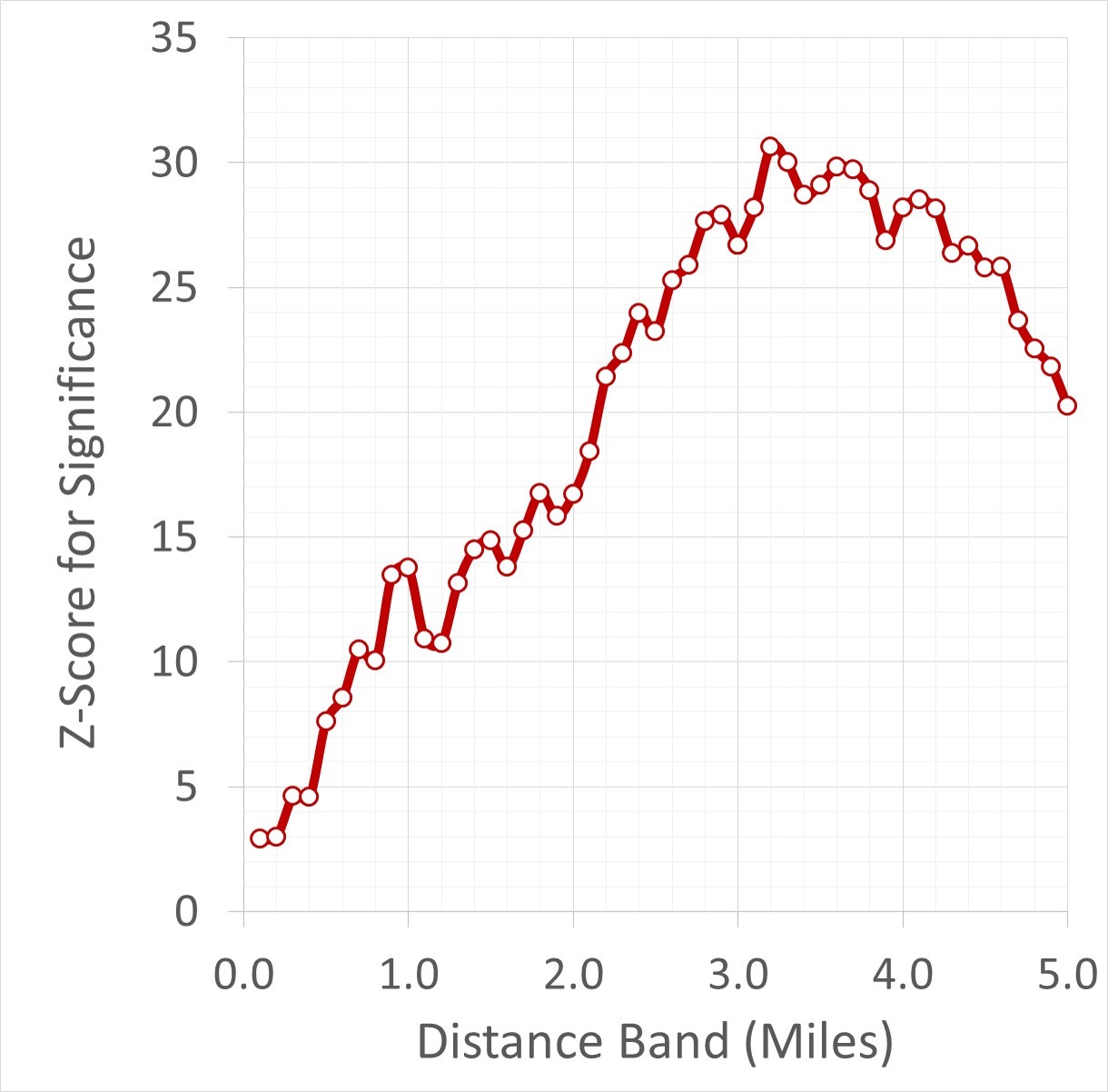


**Figure S1.** Incremental Spatial Autocorrelation to Determine Clustering Distance Band.

Legend: Graph of Z-Scores for Significance of Incremental Autocorrelation. Based on standard geographic analytic techniques, the amount of significance is analyzed for the first and maximal peaks of statistical significance. Then clustering analysis is performed using these distance bands to determine the distance within points exert influence on one another in the clustering analysis. Incremental spatial autocorrelation analysis identified the first and maximally statistically significant peaks at 1.0 and 3.2 miles.
